# Imagery – ultrasound biomicroscopy and anterior segment Optical Coherence Tomography – in the diagnosis of anterior segment pathology

**Published:** 2020

**Authors:** Mihail Zemba, Alina-Cristina Stamate

**Affiliations:** * “Dr. Carol Davila” Central Military Emergency Hospital, Bucharest, Romania; ** “Carol Davila” University of Medicine and Pharmacy, Bucharest, Romania; *** Arena Med, Bucharest, Romania

**Keywords:** anterior segment optical coherence tomography, ultrasound biomicroscopy

## Abstract

**Purpose:** The aim of this paper was to show the usefulness of imagery in better documenting the pathology of the anterior segment.

**Methods:** The article comprises clinical cases, insisting on how imagery was essential in establishing the diagnosis or the therapeutic plan.

**Results:** Lack of imagery would have made establishing a proper diagnosis much more difficult.

**Conclusions:** Although clinical examination is simple and offers a fairly good amount of information, some particular cases of anterior segment pathology need additional investigations, every method having its indications and limits.

## Introduction

The ophthalmologist can consider himself fortunate for having chosen this particular medical specialty because in most cases the doctor can readily observe the disease just by using his own eyes. Nevertheless, sometimes, with the help of modern biomicroscopes – with good illumination and different levels of magnification – ophthalmologists can have an even better view of the pathological process. This is especially true for anterior segment pathology. The cornea, the anterior chamber and the iris can be easily approached and the diagnosis rarely needs additional investigations. However, there are clinical situations when the ophthalmologist has doubts and certain imaging investigations may help him.

## Ultrasound biomicroscopy (UBM)

The use of high frequencies, with a range between 20 to 100 MHz, has improved the resolution of ultrasound images and allows the visualization of the cornea and anterior segment. The first device for clinical use was introduced by Devlin and colleagues from the laboratory of Stuart Foster at the University of Toronto and the authors named this examination method ultrasound biomicroscopy [**1**,**2**]. Limitations in current clinical imaging revealed the need to provide the maximum achievable resolution while also making it possible for the ultrasound beam to penetrate the ocular structures. The greater the frequency, the greater the resolution. For instance, for a 60 microns resolution, an operating frequency of 60 MHz is needed. The price to be paid for the increase in resolution is the loss of penetration. Human tissues have an ultrasound attenuation coefficient that increases with frequency. A frequency of 10 MHz allows a penetration of 50 millimeters, but, for 60 MHz, the penetration is only 5 millimeters.

UBM is suitable for the examination of a wide range of diseases affecting the structures within the penetration limits of this technique. 

UBM has been helpful in uncovering the mechanism of angle-closure glaucoma, due to its ability to show all the angle components and also the ciliary body. It also made it possible to study the behavior of the iris under different illumination conditions [**3**,**4**].

In trauma cases, UBM can reveal the cyclodialysis cleft even when the anterior chamber is shallow and it is not obvious with gonioscopy. UBM can make the state of the anterior chamber apparent under traumatic opacities and detect and assess the position of foreign bodies that are difficult to view using conventional techniques [**6**].

The evaluation of iris tumors can be improved by using UBM, especially in the case of angle tumors, where it can provide measurements of the tumors, including the height, that can be used for follow-up [**7**].

UBM can assess corneal and conjunctival tumors, along with the depth of the conjunctival, limbal and corneal lesions. This is especially useful for corneal involvement, where UBM can determine the thickness of the tumor and which corneal layers are involved [**8**].

UBM is very useful in assessing the pathology behind the iris – malposition of the intraocular lens, iris cysts and ciliary body tumors, but this is beyond the scope of this article.

Optical coherence tomography (OCT) is a high-resolution imaging technique that uses low coherence interferometry to obtain axial resolution in the range of 3-20 microns. Anterior segment optical coherence tomography (AS-OCT) was first demonstrated in 1994 by Izzat, using light with a wavelength of 830 nm, used mainly for the posterior pole examination. However, this wavelength does not have good penetration through the sclera, whereas the wavelength of 1310 nm allows deeper penetration and imaging of the anterior chamber, including the angle [**9**]. 

AS-OCT can be useful in the diagnosis and follow-up of different corneal diseases: dystrophies, degenerations, inflammatory pathology – corneal infiltrations, ulcers, scars. For corneal ulcers, the depth and extent obtained by AS-OCT is more accurate to that of anterior segment photography [**10**,**11**].

AS-OCT provides different tools for measuring structures of the anterior segment: accurate measurements of the angle, calipers that can measure anterior chamber depth and width and also corneal thickness. In keratoconus management, AS-OCT can document the corneal thickness and also provide good imaging of intracorneal rings [**12**].

In corneal refractive surgery, AS-OCT can measure corneal thickness, assess the flap thickness and the residual stromal bed.

In corneal transplantation, AS-OCT provides images of the host-graft junction and of retrocorneal membranes [**13**]. In endothelial keratoplasty, AS-OCT can visualize and monitor the graft attachment.

In glaucoma, AS-OCT provides direct visualization of the anterior chamber angle. Many studies showed that AS-OCT measurement of the angle is reliable and correlated to gonioscopy [**14**]. It can be used to distinguish between appositional and synechial closure, and it can also assess the result of laser iridotomy – showing whether iridectomy has increased the depth of the anterior chamber and the width of the angle [**15**]. AS-OCT has a role in the visualization of the anterior segment anatomy when suspecting malignant glaucoma and can also be used in the evaluation of the filtration bleb after trabeculectomy.

## Methods and results

**Case 1**

**Fig. 1:**
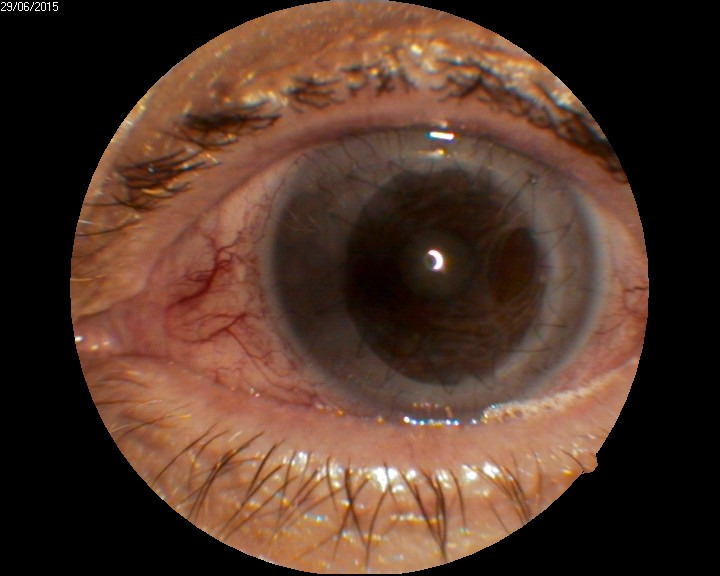
Retrocorneal membrane

We presented the case of a patient who underwent a penetrating keratoplasty for bullous keratopathy 30 days before admission. A retrocorneal membrane covered all the surface behind the graft, apart from a small region, situated temporally. There was a distance of about 1,5 millimeters between the cornea and the membrane and the same distance between the membrane and the iris (**Fig. 1**).

**Fig. 2:**
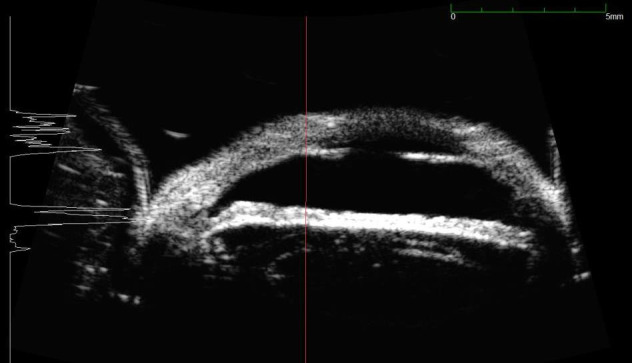
UBM – retrocorneal membrane

UBM emphasized that the retrocorneal membrane arose at the graft-host junction and covered the entire circumference, except for a small temporal region. The thickness of the membrane appeared to be 10-15% of that of the cornea (**Fig. 2**).

**Fig. 3:**
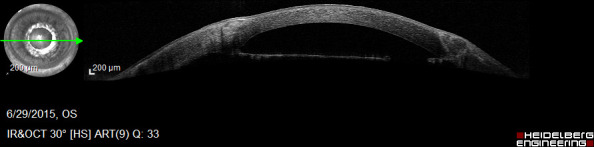
AS-OCT Retrocorneal membrane

AS-OCT showed the same modifications, but better assessed the thickness of the very thin membrane (**Fig. 3**).

**Fig. 4:**
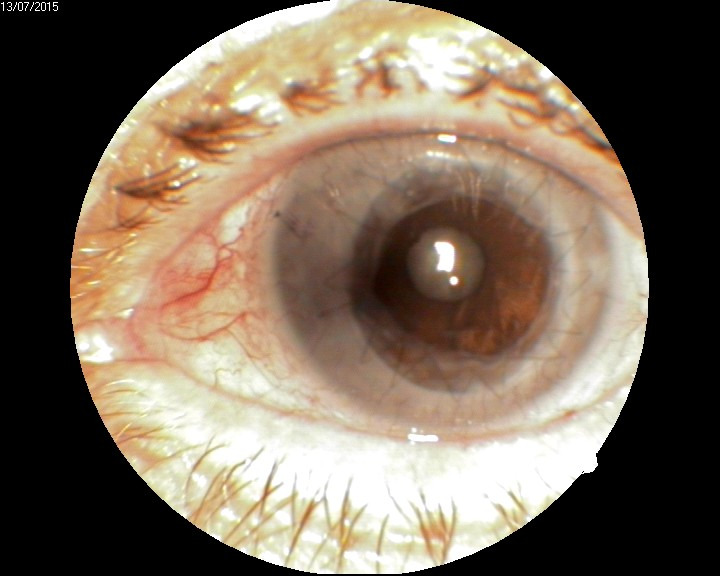
Penetrating keratoplasty after retrocorneal membrane excision

The diagnosis of retained host Descemet’s membrane was established and surgical removal was performed with good functional results (**Fig. 4**).

**Case 2**

**Fig. 5:**
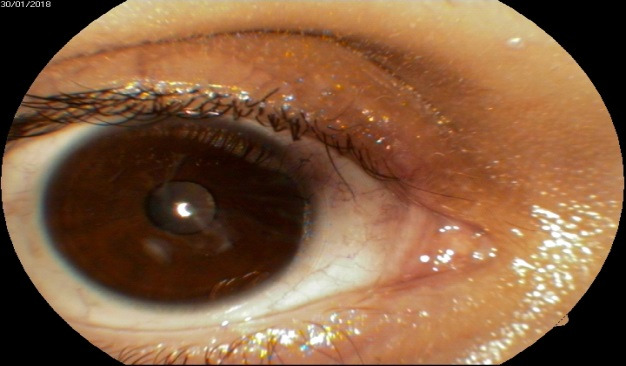
Intracorneal and intraocular glass foreign body

The second case was that of a patient who suffered a car accident and had multiple glass foreign bodies removed from his cornea in the emergency department. The patient was then referred to our clinic for a deep corneal foreign body, as well as for an intraocular foreign body (**Fig. 5**).

**Fig. 6:**
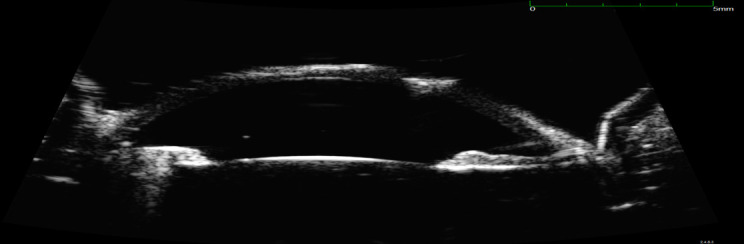
UBM – corneal and intraocular foreign body

UBM provided information regarding the location of the intraocular foreign body, which was without contact with the iris. The foreign body was removed through a corneal incision with no corneal, iris or lens damage. AS-OCT could not obtain a good view of the angle (**Fig. 6**).

**Fig. 7:**
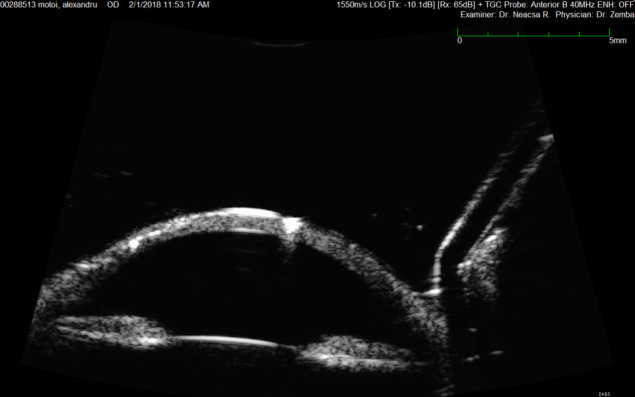
UBM – corneal foreign body

Regarding the corneal foreign body, UBM showed a hyperreflective lesion in the corneal stroma; it was difficult to assess how deep the foreign body was (**Fig. 7**).

**Fig. 8:**
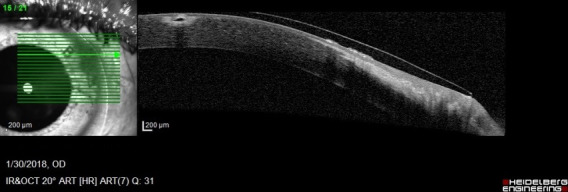
AS-OCT – corneal foreign body

AS-OCT showed a hyperreflective area with a shadow effect in the anterior stroma, quite superficially; it was removed without complications (**Fig. 8**).

**Fig. 9:**
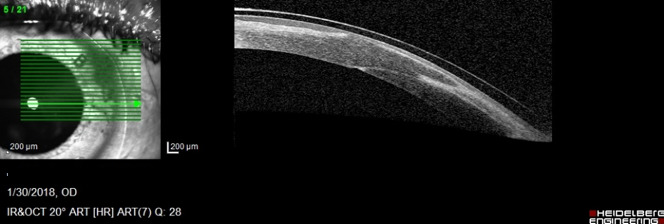
AS-OCT: corneal scars and corneal trajectory of the intraocular foreign body

AS-OCT better assessed the corneal scars and the corneal trajectory of the intraocular foreign body, information impossible to obtain by using UBM (**Fig. 9**).

**Case 3**

**Fig. 10:**
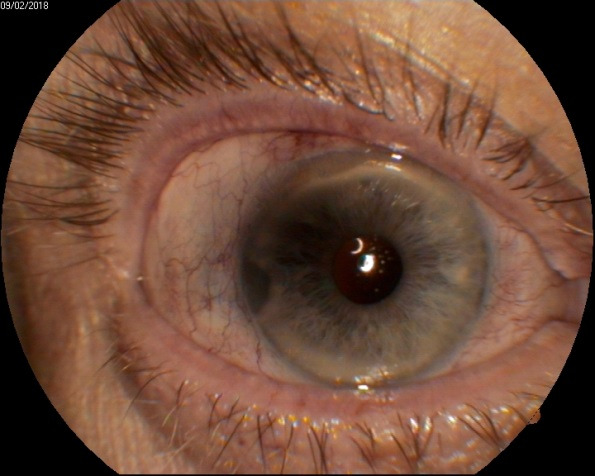
Pigmented iris lesion

A glaucoma patient who underwent trabeculectomy and phacoemulsification with intraocular lens implantation 12 months before in another clinic, came for a pigmented lesion that had appeared 6 months before and seemed to have become darker and to have increased in dimensions (**Fig. 10**). The lesion was pigmented, near the angle, about 2 millimeters in diameter, between the 8 and 9 o’clock positions. It was difficult to assess the elevation using the biomicroscope due to the reflection of light in the peripheral cornea. Gonioscopy showed a pigmented lesion, which did not appear prominent.

**Fig. 11:**
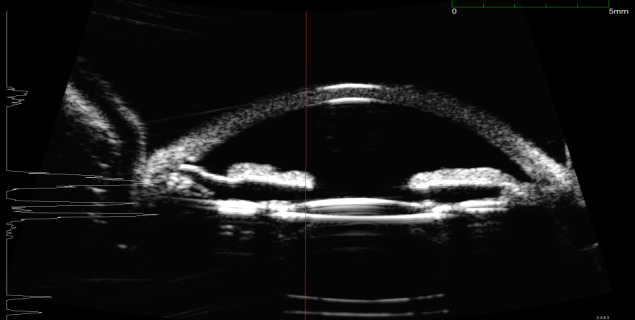
UBM – loss of substance at the level of the lesion

UBM was performed to evaluate the depth and the extension of the lesion, but surprisingly it showed that at the level of the lesion the thickness of the iris was significantly reduced (to more than 70%) (**Fig. 11**).

Most probably, the anterior surface of the iris had actually been removed with the phaco tip during phacoemulsification and there was a progressive atrophy of the remaining stroma, which allowed a better visualization of the intensely pigmented posterior layer of the iris.

**Case 4**

**Fig. 12:**
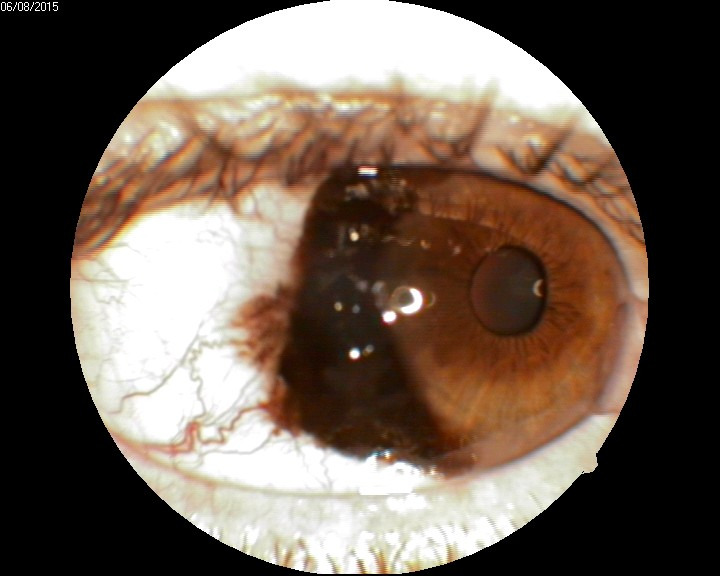
Pigmented lesion of the conjunctiva

In this case, the patient presented with a prominent, pigmented lesion of the bulbar conjunctiva, which also involved the limbus and the cornea (**Fig. 12**).

**Fig. 13:**
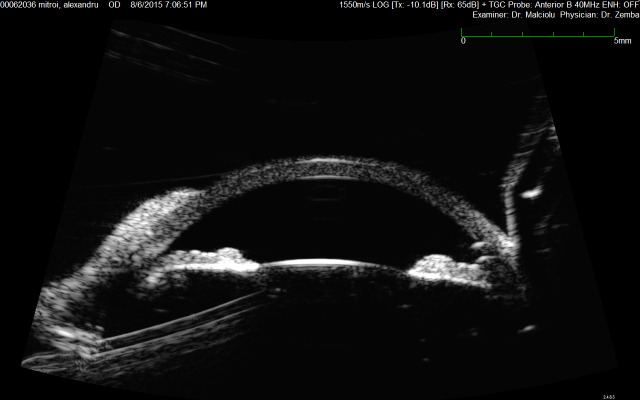
UBM – corneo-conjunctival lesion

UBM showed a hyperreflective lesion at the level of the limbus and cornea; there was a line of demarcation between the lesion and the cornea. There was no intraocular extension of the lesion, the anterior chamber angle being free (**Fig. 13**). 

**Fig. 14:**
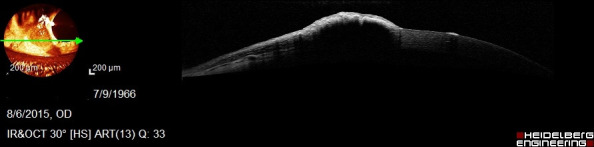
AS-OCT – corneo-conjunctival tumor

AS- OCT could not assess the structures beneath the tumor that was opaque to light (**Fig. 14**).

## Conclusion

As it can be seen, although the lesions were very accessible to direct visualization, the comprehensive assessment requires imagistic methods. Both the UBM and the AS-OCT have advantages, but also limitations. UBM can examine through opaque media – pigmented lesion, as in case 4 -, corneal scars, corneal edema. It can also visualize retroirian structures. However, it is difficult to obtain good quality images, the procedure is time-consuming and needs topical anesthesia; it is a contact method, not very pleasant for the patient and sometimes corneal erosions may occur. AS-OCT is a non-contact method; the images are taken very quickly and it is easier to obtain good quality images. A follow-up can be achieved more accurately than with UBM, because some anatomical structures – nevi, vessels – can be used as landmark points. The method is more accurate in assessing corneal lesions, but only in clear corneas. The main disadvantage is that it cannot examine through opaque media – tumors, scars, pterygium – or behind the iris.
